# Liquid-phase characterization of molecular interactions in polyunsaturated and n-fatty acid methyl esters by ^1^H low-field nuclear magnetic resonance

**DOI:** 10.1186/s13068-015-0280-5

**Published:** 2015-07-07

**Authors:** Nitzan Meiri, Paula Berman, Luiz Alberto Colnago, Tiago Bueno Moraes, Charles Linder, Zeev Wiesman

**Affiliations:** The Phyto-Lipid Biotechnology Lab, Departments of Biotechnology, Energy and Environmental Engineering, Ben-Gurion University of the Negev, P.O. Box 653, Beer-Sheva, 84105 Israel; Embrapa Instrumentação, Rua 15 de Novembro 1452, São Carlos, SP 13560-970 Brazil; Instituto de Física de São Carlos, Universidade de São Paulo, Av. Trabalhador Sao-Carlense 400, São Carlos, SP 13566-590 Brazil; Zuckerberg Center for Water Sciences and Technology and Department of Biotechnology, Ben-Gurion University of the Negev, P.O. Box 653, Beer-Sheva, 84105 Israel

**Keywords:** ^1^H low-field nuclear magnetic resonance relaxometry, Biodiesel physical properties, Fatty acid methyl esters, Melting point, Molecular packing, Segmental motion, Translational motion

## Abstract

**Background:**

To identify and develop the best renewable and low carbon footprint biodiesel substitutes for petroleum diesel, the properties of different biodiesel candidates should be studied and characterized with respect to molecular structures versus biodiesel liquid property relationships. In our previous paper, ^1^H low-field nuclear magnetic resonance (LF-NMR) relaxometry was investigated as a tool for studying the liquid-phase molecular packing interactions and morphology of fatty acid methyl esters (FAMEs). The technological potential was demonstrated with oleic acid and methyl oleate standards having similar alkyl chains but different head groups. In the present work, molecular organization versus segmental and translational movements of FAMEs in their pure liquid phase, with different alkyl chain lengths (10–20 carbons) and degrees of unsaturation (0–3 double bonds), were studied with ^1^H LF-NMR relaxometry and X-ray, ^1^H LF-NMR diffusiometry, and ^13^C high-field NMR.

**Results:**

Based on density values and X-ray measurements, it was proposed that FAMEs possess a liquid crystal-like order above their melting point, consisting of random liquid crystal aggregates with void spaces between them, whose morphological properties depend on chain length and degree of unsaturation. FAMEs were also found to exhibit different degrees of rotational and translational motions, which were rationalized by chain organization within the clusters, and the degree and type of molecular interactions and temperature effects. At equivalent fixed temperature differences from melting point, saturated FAME molecules were found to have similar translational motion regardless of chain length, expressed by viscosity, self-diffusion coefficients, and spin-spin (*T*_2_) ^1^H LF-NMR. *T*_2_ distributions suggest increased alkyl chain rigidity, and reduced temperature response of the peaks’ relative contribution with increasing unsaturation is a direct result of the alkyl chain’s morphological packing and molecular interactions.

**Conclusions:**

Both the peaks’ assignments for *T*_2_ distributions of FAMEs and the model for their liquid crystal-like morphology in the liquid phase were confirmed. The study of morphological structures within liquids and their response to temperature changes by ^1^H LF-NMR has a high value in the field of biodiesel and other research and applied disciplines in numerous physicochemical- and organizational-based properties, processes, and mechanisms of alkyl chains, molecular interactions, and morphologies.

**Electronic supplementary material:**

The online version of this article (doi:10.1186/s13068-015-0280-5) contains supplementary material, which is available to authorized users.

## Background

Diesel fuel has a vital function in the transportation sector, yet its combustion emits greenhouse gases and it is a finite resource; a cost-effective renewable substitute should have equivalent fuel efficiency, small net carbon emission, and be readily available worldwide. A potentially attractive alternative to fossil fuel is the use of plant oils—biodiesel [1]. Biodiesel is defined as mono-alkyl esters of long-chain fatty acids (FAs), offering a viable alternative to petroleum-based diesel fuel. Biodiesel is non-toxic, degrades four times faster than diesel, and its blending with diesel fuel increases engine efficiency. It also does not produce net greenhouse effects and is safer in storage due to its high flash point [2]. For these and other reasons, biodiesel production has gradually grown in recent years, raising the need for new rapid and cost-effective analytical tools and technologies for developmental characterization and quality control. ^1^H low-field nuclear magnetic resonance (LF-NMR) holds good potential in the fuel industry with many applications including determination of physical, chemical, structural, and dynamic properties.

In our previous work [3], the molecular packing of methyl oleate (18:1) in its liquid phase was studied. The results from X-ray, LF-NMR diffusiometry, and high-field (HF)-NMR measurements were rationalized for the first time by a given liquid-phase packing model of fatty acid methyl esters (FAMEs; biodiesel). The developed model for 18:1 proposed that liquid FAMEs have short range order, where molecules arrange in a head-to-head conformation due to polar interactions, and their aggregate morphology retains a quasi-smectic liquid order mainly due to weak intermolecular interactions between two adjacent backbone molecules. The two molecule chains arrange longitudinally, and alternately to make an interdigitated structure, where in the same lateral plane, the ester groups of one molecule and the terminal methyl groups of the neighboring molecule are aligned side by side, similar to FAs [4, 5]. The translational movement of FAMEs, on the other hand, was found to differ from that of FAs, which are mostly dimerized due to head group hydrogen bonding. While the FA dimer is the basic unit of inter- and intra-molecular movements [4, 5], FAME molecules diffuse as monomers.

Biodiesel is a mixture of different FAMEs, with 18:1 as one of the main components, and each component affects the biodiesel properties as a function of its relative concentration. Therefore, to fully characterize biodiesel properties, it is necessary to further investigate different FAMEs that vary in chain lengths and number of double bonds.

Very little research has been performed on the liquid-phase molecular organization of FAMEs. In general, the physical properties of FAs and their biodiesel derivatives are largely determined by the length of the hydrocarbon chain, the degree of unsaturation, and the effect of molecular packing. In the fully saturated compounds, the hydrocarbon chain is highly flexible with free rotation around each carbon–carbon bond. The most stable conformation is the completely stretched arrangement, wherein the steric hindrance of neighboring atoms is minimized. This conformation allows tight packing in nearly crystalline arrays, stabilized by van der Waals force interactions between the atoms of neighboring molecules. In unsaturated hydrocarbons, chain bending occurs due to *cis* double bonds. The intermolecular interactions of molecules with alkyl chains having several *cis* bond bends are weaker than molecules with only one *cis*, because of less compact packing compared to one *cis* bond bend or a fully saturated hydrocarbon. These loosely ordered arrays of unsaturated molecules have lower melting points than monounsaturated molecules of the same chain length, because less thermal energy is needed for overcoming molecular interactions [6].

Matsuzawa et al. [7] studied different molecular packing densities and suggested that the existence of aggregate clusters most likely determines the liquid properties of FAs such as density and fluidity. It is feasible to assume that this is similar for FAMEs; hence, the liquid morphological structure of these materials will affect the physicochemical properties of the biodiesel including viscosity, density, fluid dynamics, and low temperature operability. These properties are of great importance to the field of biodiesel and each is based on different liquid characteristics. For example, viscosity, defined as a liquid’s resistance to flow, is a function of the intermolecular forces of attraction within a liquid. Density, on the other hand, defined as the mass per unit volume of a material, depends on how tightly the molecules are packed together. The former rely on interactions between one molecule to its neighbor, while the latter depends on the conformation of a molecule and its packing density properties.

It has been shown that ^1^H LF-NMR spin-spin (*T*_2_) relaxometry can be applied to differentiate between morphological populations in complex systems [8–12]. Still, there isn’t a certainty about the origin of triacylglycerol peaks in ^1^H LF-NMR relaxation time distributions. It has been suggested that the bimodal *T*_2_ distribution of liquid tricaprin is due to inhomogeneous relaxation rates for the protons along the side chains or inhomogeneous organization of the triacylglycerols in the liquid with intermolecular interactions [13]. In our previous work [3], these hypotheses were considered regarding oleic acid (18:1 acid) and methyl oleate (18:1), two similar alkyl chain molecules with different head groups, and it was suggested that the two peaks are the result of two distinct mobility populations of the protons on the chain.

In the present study, in order to characterize FAME aggregate structures and how they influence viscosity, liquid density, and temperature effects, we focused on further exploring the assignment of the bimodal peaks in ^1^H LF-NMR *T*_2_ distributions for different FAME molecules, with similar head groups but different alkyl chain lengths (10 to 20 carbons) and degrees of unsaturation (0, 1, 2, and 3 double bonds). Our objective was to study the relationship between molecular organization versus segmental and translational movements of different FAMEs in their pure liquid phase using ^1^H LF-NMR relaxometry and supporting advanced technologies, including X-ray diffraction, ^1^H LF-NMR diffusiometry, and ^13^C HF-NMR.

## Results and discussion

### ^1^H LF-NMR *T*_2_ distributions of FAMEs at 313 K

Biodiesel is a complex mixture of FAMEs with different lengths, degrees of unsaturation, and composition. It is therefore important to understand the molecular organization versus segmental and translational movements of the separate FAMEs, in order to explain their behavior in the biodiesel blend. The FAMEs evaluated in this study and their literature data of melting temperatures (denoted as *T*_m_) are summarized in Table 1 [14–17]. Throughout this manuscript, FAMEs are identified by their structures.

**Table 1:** Summary of the FAMEs evaluated in this study and literature data of melting temperatures (*T*_m_s)

IUPAC name	Common name	Structure	*T*_m_ [K]
Methyl decanoate	Methyl caprate	10:0	259.5 [14]
Methyl dodecanoate	Methyl laurate	12:0	278.0 [15]
Methyl tetradecanoate	Methyl myristate	14:0	292.0 [16]
Methyl hexadecanoate	Methyl palmitate	16:0	303.0 [16]
Methyl octadecanoate	Methyl stearate	18:0	312.1 [16]
Methyl eicosanoate	Methyl arachidate	20:0	318.8 [17]
Methyl Z-9-hexadecenoate	Methyl palmitoleate	16:1	238.9 [14]
Methyl Z-9-octadecenoate	Methyl oleate	18:1	253.0 [15]
Methyl Z,Z-9,12-octadecadienoate	Methyl linoleate	18:2	238.0 [15]
Methyl Z,Z,Z-9,12,15-octadecatrienoate	Methyl linolenate	18:3	227.5 [15]

*T*_m_ melting temperature

**Fig. 1:**
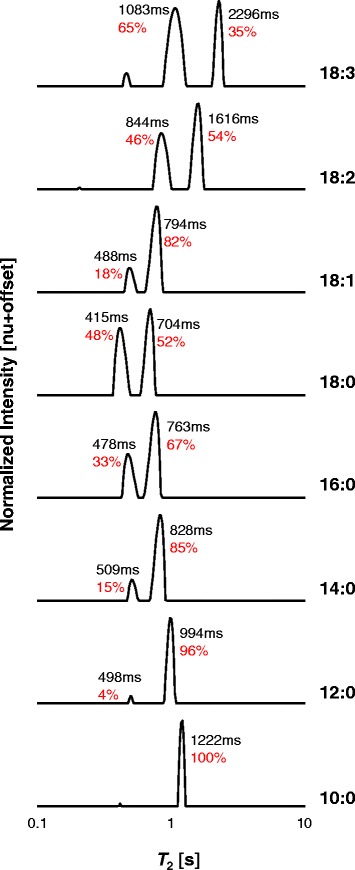
Combined ^1^H LF-NMR *T*_2_ distributions of FAMEs at 313 K. Plots are arranged by increasing chain length (*bottom* to *top*) and increasing degrees of unsaturation. The relative contributions of each peak in relation to the other peak and intrinsic *T*_2_ values are shown on each plot. FAMEs are referred by their structures

The combined ^1^H LF-NMR *T*_2_ distributions of some saturated and unsaturated FAMEs at 313 K are presented in Fig. 1. *T*_2_ distributions are arranged by increasing chain length and degree of unsaturation. Intrinsic *T*_2_ values and percentage relative contribution (RC) of the peaks are marked on each plot. All *T*_2_ distributions exhibit two distinct peaks at different *T*_2_ values and RC. These will be denoted as *P*_1_ and *P*_2_ with increasing *T*_2_ values (*T*_2,1_ and RC_1_ will therefore stand for intrinsic *T*_2_ value and RC of *P*_1_). FAME 18:3 was found to be very prone to oxidation. Almost immediately following the first measurements an additional peak at low *T*_2_ values appeared that increased over time (Additional file 1). This same event occurred with different fresh samples and at different temperatures. In this study, therefore, fresh samples were used whenever the oxidation peak exceeded an RC of 5 %, and only the two main peaks, not related to oxidation, are discussed.

As shown, each FAME exhibits a slightly different *T*_2_ distribution, which can be explained by the differences in chemical structures. For the saturated esters, with increasing chain length RC_1_ increases in relation to RC_2_ (0, 4, 15, 33, and 48 % RC_1_ for 10 to 18 carbons) and both *T*_2,1_ and *T*_2,2_ values decrease. For the unsaturated esters, with increasing number of double bonds, again RC_1_ increases in relation to RC_2_ (18, 46, and 65 % RC_1_ for 1 to 3 double bonds, respectively) whereas *T*_2_ values of both peaks increase.

Following the peak assignments we previously suggested [3]; it is proposed that FAMEs have reduced mobility with increasing chain length and saturation, and intra-molecularly more rigid parts with increasing chain length and degree of unsaturation. Interestingly, for the 18 carbon esters, when comparing the fully saturated to monounsaturated, RC_1_ decreases to a greater extent (48 versus 18 % RC_1_ for 18:0 and 18:1, respectively). This may suggest different molecular organizations and/or type of interactions for the saturated versus unsaturated FAMEs, since the morphology of the alkyl chains, intermolecular interactions, and absolute temperature of measurement contribute to the ^1^H LF-NMR *T*_2_ distributions of FAMEs.

In our previous work, we compared *T*_2_ distributions of 18:1 and 18:1 acid relative to the temperature of melting of each compound, and several similarities were suggested for the peaks of each standard, which strengthened their assignment to two distinct mobility populations of the protons on the chain. In the case of 18:1 at 288 K, the short *T*_2_ peak was assigned as the protons on the chain backbone and the larger *T*_2_ peak as the protons on both ends of the chain. Adam-Berret et al. [13] suggested that liquid triglycerides with three similar fully saturated alkyl chains have comparable behavior for the same difference from their melting temperature and that the effect of chain length was attenuated with this representation. A similar observation was suggested for different chain length liquid alkanes [18]. This similar behavior is the result of comparable structures and type of interactions between alkyl chains.

Prior to melting, the atoms in a solid have restricted molecular motion and are confined to vibrating about their mean positions within the lattice/morphology structure of the solid. By increasing the temperature of the solid matter, the amplitude of the molecular vibrations increases, until at a certain temperature, intermolecular bonds within the solid break, allowing for bond rotation, and the molecules become free to rotate and translate within the liquid volume. The temperature of transition for a specific substance is the melting point. This is determined by the strength of a crystal lattice, which in turn is controlled primarily by four factors: the nature and number of intermolecular forces, molecular symmetry and packing efficiency, and the conformational degrees of freedom of a molecule [19].

Following this approach, in order to gain a deeper understanding of the molecular organization and rotational and translational motions of saturated FAMEs in the liquid phase, additional measurements in this work include comparisons according to melting temperatures of each FAME material. Unsaturated FAMEs, on the other hand, have different intermolecular interactions, molecular freedom, and packing, leading to more complicated molecular structures, and rotational and translational motions, which cannot be standardized comprehensively by melting point. These will therefore be compared at absolute temperatures of measurement.

### X-ray measurements

In order to explore the molecular arrangement of the saturated FAMEs in this present study, the long- and short-range spacings were determined using small angle X-ray scattering (SAXS) and X-ray diffraction (XRD), respectively, close to their melting points at *T*_m_ + 15 K (Table 2). The X-ray diffraction spectra acquired resemble those of 18:1 acid and 18:1 methyl ester, as previously reported [3]. Two bands at around 0.14 and 0.03 nm^−1^ were observed, which give a measure of the spacing between adjacent molecules (short-range spacing) and long-range spacing of the plane as a result of head groups packing of the aligned molecules, respectively. Due to technical specifications of the XRD instrument, short-range spacing measurements were only available above ambient temperature; therefore measurements of short (10 and 12 carbons) saturated FAMEs, whose *T*_m_ + 15 K are below 298 K were performed at 298 K. SAXS showed no peaks for 10:0 at *T*_m_ + 15 K. It was thus measured at *T*_m_ + 5 K.

**Table 2:** Short- and long-range spacing of saturated FAMEs measured at *T*_m_ + 15 K

FAME structure	Short spacing [nm]	Long spacing [nm]
10:0	0.459^a^	1.70^b^
12:0	0.461^a^	2.08
14:0	0.459	2.27
16:0	0.462	2.53
18:0	0.455	2.64
20:0	0.457	2.83

*T*_m_ melting temperature

^a^Measurements performed at ambient temperature

^b^Measurement performed at *T*_m_ + 5 K

All the FAMEs measured exhibited similar short-range spacing. As anticipated, for the saturated FAMEs, longer long-range spacings were found with increasing chain length. The long-range spacing for 10:0, however, was expected to be approximately half that of 20:0. According to Table 2, this was not the case. This same occurrence was previously presented for the long-range spacing of 8:0 acid [7] and was explained as 8:0 acid molecules being only partly interdigitated compared to 18:0 acid molecules, which are completely interdigitated. We believe that the same arrangement applies also for the FAMEs in hand, since even though the head group is different, the structure of the tails is similar.

Above melting temperature, and in accordance with our previous work on 18:1 [3], the results are explained in that the studied FAMEs possess a liquid crystal-like structure, consisting of randomly aggregated liquid clusters with void spaces between them. This kind of microstructural organization can be the result of structural memory coming from the solid structure of FAMEs molecules, where polar interactions exist between heads and van der Waals forces are between two adjacent backbones forming an interdigitated structure. Evidence for this microstructural arrangement can be viewed by differences in density (Table 3). The reported data was compiled from the studies by Pratas et al. [20, 21] for an absolute temperature of 313 K. With increasing temperature, the density of saturated and monounsaturated FAMEs decreases linearly, as suggested by Knothe and Steidley [22].

**Table 3:** Densities, *ρ*, of some FAMEs measured at 313 K (compiled from the literature)

FAME structure	*ρ* [kg/m^3^]
10:0	856.0 [20]
12:0	853.9 [20]
14:0	852.2 [20]
16:0	850.8 [20]
18:0	849.8 [20]
16:1	853.8 [21]
18:1	859.5 [20]
18:2	871.5 [20]
18:3	887.0 [20]

*ρ* density

According to this data, for saturated FAMEs, density slightly decreases with increasing chain length. For the same number of carbons, density increases with increasing number of double bonds. These density measurements suggest, contrary to our expectation from molecular translational studies, that 18:3 molecules are the most closely packed, while saturated FAMEs are the most loosely packed. Iwahashi and Kasahara [23] found similar behavior for saturated and monounsaturated FAs and have explained this discrepancy by the form of cluster aggregation. They suggested that saturated molecules aggregate tightly to make rigid clusters. These clusters form a liquid morphology with many void spaces between them leading to an overall lower liquid density and, consequently, a large apparent molar volume. Unsaturated molecules, on the other hand, form similar but softer clusters and can aggregate closer together to form smaller and fewer void spaces, leading to increased density and molar volume.

The same holds for the FAMEs in this study. Apparently, chain length can have the same effect on density, where the shorter the chain, the less number of interactions and the softer the cluster leading to increased packing density of the cluster and liquid density. Interestingly, the decrease in density with increasing chain length is not uniform. For the saturated compounds at 313 K, density decreases by 2.1, 1.7, 1.4, and 1.0 kg/m^3^ for 10 to 18 carbons, respectively. This is true also for other temperatures according to Knothe and Steidley [22] and may be explained by the degree of interdigitation of rods inside the clusters as previously discussed.

### Self-diffusion coefficients

**Fig. 2:**
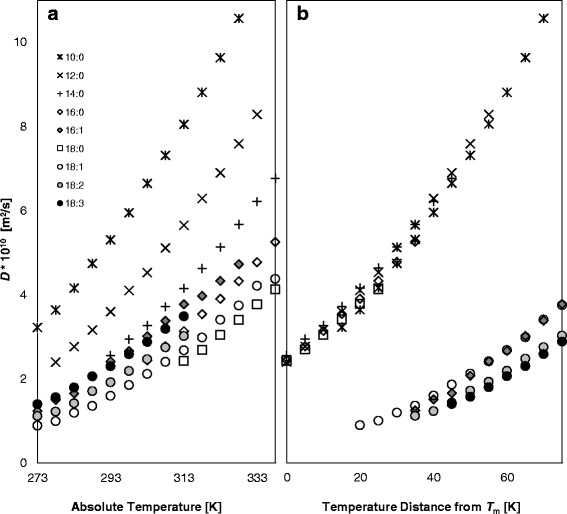
Self-diffusion coefficients, *D*, of FAMEs in response to temperature. *D* measurements are compared at **a** absolute temperature and **b** temperature distances from each sample’s melting point (*T*_m_) for all FAMEs. FAMEs are referred to by their structures. For the absolute temperatures (**a**), diffusion increases with decreasing chain length and for the same chain length increasing number of double bonds. For the normalized temperatures (**b**), saturated FAMEs exhibit the highest and almost similar translational movement, followed by the monounsaturated FAMEs (16:1, 18:1), the di- (18:2), and tri-unsaturated (18:3) ones. The conflicting trends can be traced back to the change in morphology and translational motion above the melting point

The effect of the chemical structure of FAMEs on their translational movement is shown in Fig. 2a, b for measurement at a single temperature that was above the melt points for all the FAMEs and for normalized temperature differences according to the *T*_m_ of each FAME, respectively. Interestingly, these results show conflicting trends that can be traced back to the change in morphology and translational motion above the melting point. The self-diffusion coefficient, *D*, is related to a fluid’s viscosity from the Stokes-Einstein equation. From the physicochemical point of view, viscosity is related to the resistance of a molecule to move/slide relative to another molecule. Therefore, viscosity must be closely correlated with the structural parameters of the fluid particles [24].

In Fig. 2a (the same temperature for all FAMEs), the shorter the chain of the fully saturated FAME, the faster it diffuses. This can be explained by fewer intermolecular bonds and by the partly interdigitated structure of the shorter FAMEs. For the same chain length, the more double bonds, the larger the *D*. These results correlate very well with the *T*_2_ relaxation distributions presented in Fig. 1. It is well known that kinematic viscosity increases with chain length and with increasing degree of saturation [15]. The effect of unsaturation has been attributed to interference of the double bonds with the molecules’ ordered structure by adding kinks to the chain. Ramirez-Verduzco et al. [25] suggested that coil-like *cis* configuration hinders the interactive approach of the double bond carbon atoms with the double bond carbons of neighboring molecules. This means that the translational movement of the unsaturated FAMEs increases with the number of double bonds.

When comparing the translational motion of the FAMEs at similar distances from their melting point (Fig. 2b) different trends are observed. Apparently all saturated FAMEs, regardless of chain length, exhibit almost similar translational movement. This can be rationalized by their similar structures and interactions, resulting in similar aggregate morphologies. The increase in melting points with chain length (Table 1) is accounted for by the increase in the number of van der Waals interactions. Therefore, at similar distances from the melting point, the differences in translational motion are attenuated by the relative quantities of thermal energy. In addition, saturated FAMEs exhibit the highest translational movement, followed by the monounsaturated FAMEs (16:1, 18:1) and then by the di- (18:2) and tri-unsaturated (18:3) ones. As suggested before, when comparing FAMsEs with different degrees of unsaturation, several additional variables need to be considered that can explain the opposite trend in *D*, including temperature, type, and number of interactions, and aggregate morphologies for different alkyl chain configurations.

### Segmental motion

**Fig. 3:**
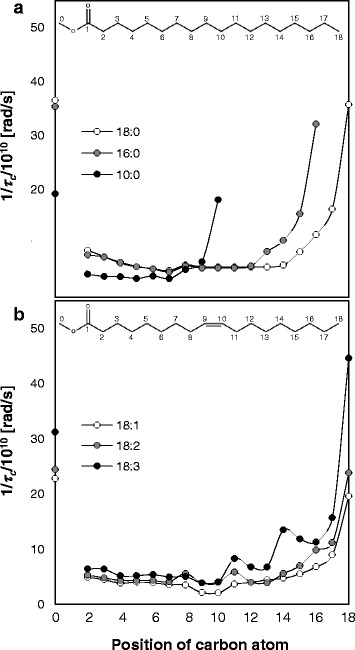
Segmental motion of some FAMEs. Segmental motion through the reciprocal of the effective correlation time, 1/*τ*_c_, of the carbon atoms at different positions of **a** saturated FAMEs (10:0, 16:0, and 18:0) at *T*_m_ (melting point) +15 K and **b** unsaturated FAMEs (18:1, 18:2, and 18:3) at 298 K. FAMEs are referred to by their structures. The structures of 18:0 and 18:1 in **a** and **b**, respectively, along with designation of carbon numbers are shown for reference

Segmental motion through the reciprocal of the effective correlation time, 1/*τ*_c_, of each carbon can be calculated from the longitudinal relaxation time, *T*_1_, measured by ^13^C HF-NMR. The 1/*τ*_c_ values of some saturated and unsaturated FAMEs were measured at *T*_m_ + 15 K and 298 K, respectively (Fig. 3a, b). *T*_1_, is likely to be correlated to the movement of the carbon atoms, i.e., segmental motion (specifically, rotational tumbling and to a lesser extent translational and internal motion) in the molecule. Assignment of ^13^C chemical shifts to the appropriate peaks was performed according to the literature [26]. The 1/*τ*_c_ value for C1 is not shown since the carbonyl carbon has no covalent bonded protons. According to the decrease in 1/*τ*_c_ values from both ends towards the center of the chain, it is suggested that both the tail and head in all FAMEs have a higher freedom of movement compared to the interior chain. Although some degree of polar interactions exists between two heads, 1/*τ*_c_ values suggest that this interaction is very weak.

According to Fig. 3a, the longer the chain length, the more rigid carbons there are in the interior part of the FAME molecule chain. These results fit very well with the data presented in the literature [27] for the segmental motion of 9:0 acid and 18:0 acid. This can be rationalized by an increase in van der Waals interactions with increasing chain length, as suggested also by the molecular organization as viewed from the X-ray results (partial versus full interdigitated structure for short- and long-chain FAMEs, respectively). The lower 1/*τ*_c_ values for all carbons in 10:0 compared to 16:0 and 18:0 can be attributed to the large difference in the absolute temperature of measurement of the three (273 K for 10:0 versus 318 and 328 K for 16:0 and 18:0, respectively).

For the unsaturated FAMEs at an absolute temperature of 298 K, the rotational movement of both ends of the chain increases with number of double bonds. This correlates with the self-diffusion coefficient of unsaturated FAMEs at absolute temperatures (Fig. 2a), since as suggested in the literature, the segmental movements at the end and near the end of the molecule are probably most important for the FAME molecules to find the spaces for their translational diffusion [3, 28]. Segmental motion of the head and tail is facilitated due to the bending of the molecule, which leads to reduced van der Waals interactions and higher degree of freedom. The motion of the double bond carbons, on the other hand, is considerably restricted (Fig. 3b). Pi et al. [29] stated that the movement and bending of alkyl chain of 18:2 acid from C9 to C13 is more regulated compared with that in the alkyl chain of 18:1 acid due to the presence of an additional C = C bond. The decrease in segmental motion at the double bond position can therefore be attributed to the larger energy barrier to bond rotation as a result of the double bonds and stronger intermolecular interactions between the *π* electrons. This implies increased rigidity with an increased number of double bonds as previously suggested according to the relative contribution of the peaks in *T*_2_ distributions (Fig. 1).

### ^1^H LF-NMR *T*_2_ distributions at different temperatures

**Fig. 4:**
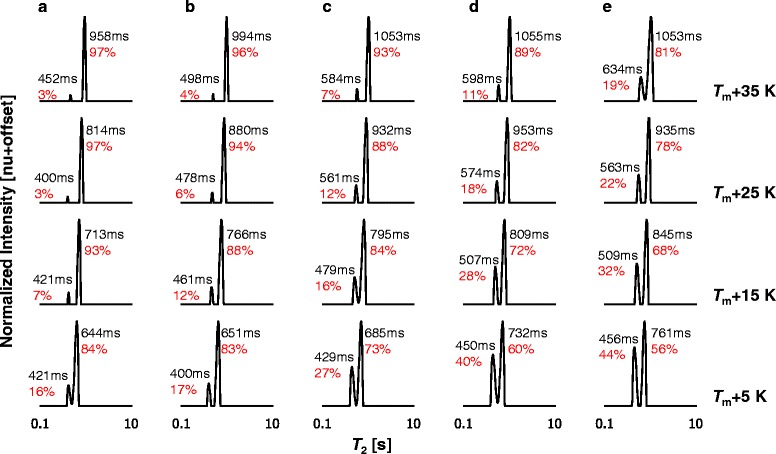
Combined ^1^H LF-NMR *T*_2_ distributions of saturated FAMEs at different temperatures. Comparison between the *T*_2_ distributions of the saturated **a** 10:0, **b** 12:0, **c** 14:0, **d** 16:0, and **e** 18:0 FAMEs at different temperature distances from their melting points (*T*_m_). FAMEs are referred to by their structures. The relative contributions of each peak in relation to the other peak and intrinsic *T*_2_ values are shown on each plot

**Fig. 5:**
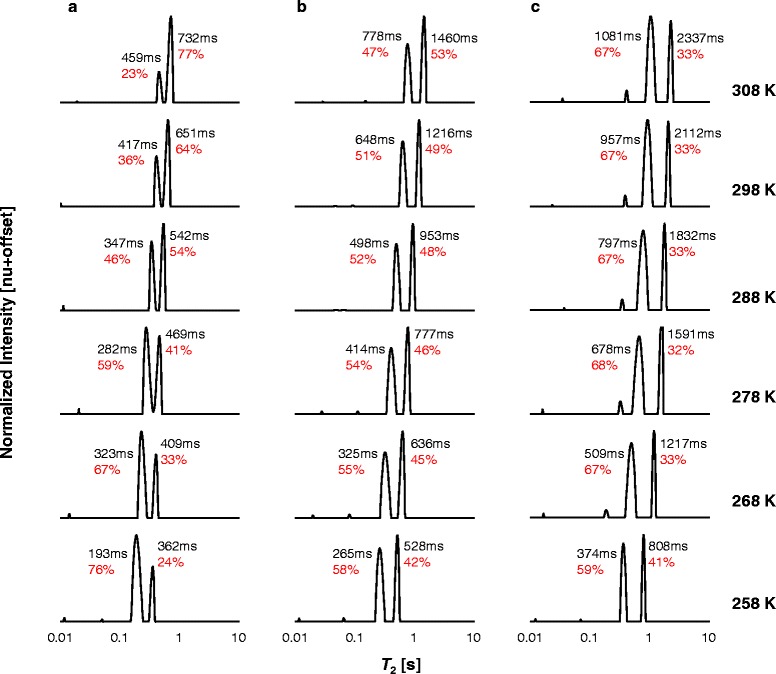
Combined ^1^H LF-NMR *T*_2_ distributions of unsaturated FAMEs at different temperatures. Comparison between the *T*_2_ distributions of the unsaturated **a** 18:1, **b** 18:2, and **c** 18:3 FAMEs at different temperatures. FAMEs are referred to by their structures. The relative contributions of each peak in relation to the other peak and intrinsic *T*_2_ values are shown on each plot

The combined ^1^H LF-NMR *T*_2_ distributions of saturated and unsaturated FAMEs are presented in Figs. 4a–e and 5a–c with increasing chain length and degree of unsaturation, respectively. For the saturated FAMEs, measurements are compared at similar temperature distances from their *T*_m_, whereas the results for the unsaturated FAMEs are shown at absolute temperatures of measurement. Intrinsic *T*_2_ values and percentage RC of each peak are marked on each plot.

In accordance with our previous work [3], for the saturated FAMEs, as temperature increases *T*_2,1_ and *T*_2,2_ shift to higher values and RC_1_ decreases in favor of RC_2_. For the saturated FAMEs, the effect of presenting results at specific distances from melting point versus one absolute temperature can be seen by comparing *T*_2_ distributions in Fig. 4a–e, at any one of the temperature distances, to Fig. 1. According to this normalization, certain constancy can be observed in *T*_2,1_ and *T*_2,2_, especially for 10:0 and 12:0, and for 14:0–18:0. RC_1_, on the other hand, increases with chain length in relation to RC_2_, as stated for the absolute single temperature comparison (Fig. 1). This increase can be explained by the addition of rigid parts of the molecule and the number of van der Waals interactions, as manifested from the segmental motion (Fig. 3a).

It was established in the past that mono-exponential *T*_2_ values of FAMEs correlate with their viscosity for the same temperature [30, 31]. Table 4 presents the mono-exponential *T*_2_s for the saturated FAMEs and dynamic viscosities, *η*, according to [20], at *T*_m_ + 5 K and at 313 K for reference. Mono-exponential *T*_2_s at 313 K were calculated from the relaxation data used for the analyses presented in Fig. 1. As shown, saturated FAMEs exhibit very similar translational motion at similar distances from melting point compared to absolute temperatures, according to the dynamic viscosity values and mono-exponential *T*_2_s, even though their melting points differ to a great extent (Table 1). These results are comparable with the self-diffusion coefficients presented in Fig. 2a, b for absolute and normalized temperatures, respectively. In addition, the mono-exponential *T*_2_s at *T*_m_ + 5 K in Table 4 resemble each other to a greater degree than *T*_2,1_ and *T*_2,2_ in Fig. 4 for the same temperatures (for 10:0 to 18:0 a *T*_2_ range of 589–617 versus 644–761 ms for mono-exponential *T*_2_ and *T*_2,2_, respectively). This suggests that the overall translational motion of saturated FAMEs is not affected solely by the mobility of the different parts of the molecules, and that the RCs of the peaks indicate other molecular parameters (e.g., molecular interactions) that also play an important part.

**Table 4:** Mono-exponential, *T*_2_, and dynamic viscosity, *η*, of saturated FAMEs at *T*_m_ + 5 K and 313 K

	*T*_m_ + 5 K		313 K	
FAME structure	*T*_2_ [ms]	*η* ^a^ [mPa · s]	*T*_2_ [ms]	*η* ^a^ [mPa · s]
10:0	589	na	1206	1.48
12:0	601	4.07	974	2.08
14:0	606	3.98	774	2.84
16:0	612	4.21	662	3.75
18:0	617	4.43	560	4.99

*na* data not available, *T*_m_ melting temperature, *T*_2_ spin-spin relaxation time, *η* dynamic viscosity

^a^Dynamic viscosity values were taken from [20]

The unsaturated FAMEs (Fig. 5a–c) also display an increase in *T*_2,1_ and *T*_2,2_ with temperature. The RCs of the peaks, on the other hand, exhibit different trends with temperature and number of double bonds. In general for the unsaturated FAMEs, RC_2_ increases with temperature in relation to RC_1_; however, this response is attenuated with increasing number of double bonds. For the temperature range 258–308 K, RC_2_ increases in the ranges 24–77, 42–53, and 33–35 % for 1–3 double bonds, respectively. The change in RC of the peaks with increasing temperature and unsaturation may reflect the number and type of weak intermolecular interactions.

In this study, our characterization of different FAME molecules by ^1^H LF-NMR relaxometry, diffusion, ^13^C HF-NMR, and X-ray methods gives a clear indication of their molecular morphology and intermolecular interactions. For the saturated molecules, the longer the chain length, the higher the melting point. This is due to their linear chain configuration, which allows for molecules to pack closely together, maximizing the number of van der Waals contacts. This is manifested by increasing RC_1_, reduced diffusion coefficient, and a decrease in the segmental motion of the backbone carbons. For the same chain length and increasing number of double bonds, melting temperatures decrease. Unsaturated molecules of *cis* configuration cannot pack as close due to bending of the chain at the double bond position, minimizing secondary interactions. As seen from their melting temperatures, they require much less energy for disordering the crystal structure and breaking intermolecular interactions to achieve molecular motion above the melt point. This leads to an increase in diffusion coefficients and segmental motion of the tail.

When going from the fully saturated to the monounsaturated 18-carbon molecule, a reduction in RC_1_ occurs in the ^1^H LF-NMR *T*_2_ distributions (Fig. 1). This can be explained as a sharp decrease in the number of van der Waals interactions due to bending of the 18:1 chain, as previously discussed. When increasing the number of double bonds, increase in π-π interactions takes place as revealed by an increase in RC_1_ as shown when going from 18:1 → 18:2 → 18:3. This can also explain the decrease with unsaturation of the RC_1_ response to increasing temperature, since it is well known that less energy (temperature) is required to break van der Waals interactions compared to π-π interactions.

These results strengthen the peak assignment suggested for the ^1^H LF-NMR *T*_2_ distributions of FAMEs, where the two peaks are the result of two distinct mobility populations of the protons on the chain affected by the molecular structure and weak intermolecular interactions, especially those between the backbone of two adjacent chains. In a previous paper [32], we presented the ^1^H LF-NMR *T*_2_ distribution of a rapeseed biodiesel sample measured at 313 K. In that study, the biodiesel sample exhibited three peaks with intrinsic *T*_2_ values of 338, 671, and 1141 ms. Given that the main constituents in this biodiesel at decreasing ratios are 18:1, 18:2, 18:3, 16:0, and 18:0, the peaks can be assigned as the average contribution according to the three regions designated a, b, and c in Additional file 2. An interesting study would be to explore the change in ^1^H LF-NMR RC of the peaks in biodiesels from different sources, storage conditions and shelf-life, and different temperatures, to study dynamic processes, melting mechanisms, and structural organizations of alkyl chains, with important applications in the development of biodiesel fuels.

## Conclusions

Both the peaks’ assignments for ^1^H LF-NMR *T*_2_ distributions of FAMEs and the model for their liquid crystal-like structure/morphology in the liquid phase, used to rationalize the assignment, were confirmed in the present work. This morphology along with the number and type of interactions and temperature effects generated differences in translational and rotational movements of the molecules, which were monitored using the presented ^1^H LF-NMR methodology. The study of morphological structures within liquids and their response to temperature changes by ^1^H LF-NMR is a powerful tool and is supported by traditional methods of characterization, such as X-ray and ^13^C HF-NMR. This new application of ^1^H LF-NMR is of potentially great interest to the field of biodiesel and to other research and applied disciplines with the potential of studying numerous physicochemical- and molecular organizational-based properties, processes, and mechanisms of alkyl chains.

## Materials and methods

### Materials

Pure samples (≥99 %) of methyl ester standards (Table 1) were purchased from Sigma Aldrich and used without further purification. These FAMEs exhibit a wide range of melting points and consequently are in different states for the same absolute temperature [33]. Some of the measurements were therefore compared according to a given temperature distance from melting point (*T* = *T*_m_ + *d*, where *T* is the actual temperature of measurement, *T*_m_ is the melting point, and *d* the temperature distance from *T*_m_). The melting temperatures used for the materials in this study are within ±2 K from the melting temperatures reported in the literature (Table 1).

### ^1^H LF-NMR relaxometry and diffusiometry

Measurements were carried out on a 20-MHz minispec bench-top pulsed NMR analyzer (Bruker Analytic GmbH, Germany), equipped with a permanent magnet, and a 10-mm temperature controlled probe head. Prior to measurement, samples were heated from 193 K for a minimum 3 h and then allowed to equilibrate inside the instrument for 5 min. All measurements were performed on liquid standards (above melting point). Receiver gain was optimized for each temperature and sample.

Determination of spin-spin relaxation time constant (*T*_2_) was performed using a Carr-Purcell-Meiboom-Gill (CPMG) pulse sequence [34, 35]. *τ* and recycle delay were between 0.4 and 1.5 s and 4 and 8 s, respectively. For all the analyses, 32 scans were accumulated and 8000 echoes were acquired. Data was acquired in magnitude mode due to better repeatability and stability of results and further analyzed using the PDCO inverse Laplace transform optimization algorithm with *a*_2_ = 0.5 as described in the literature [36].

Mono-exponential fitting of the acquired CPMG raw data was performed by SPSS software (version 15.0, SPSS Inc.) using Eq. 1:

1 $$ s(t)=w{\mathrm{e}}^{-t/{T}_2} $$

where *s*(*t*) is the acquired signal at *t* time, *w* is the pre-exponential weighting factor, and *T*_2_ is the mono-exponential relaxation time constant for transverse relaxation.

The self-diffusion coefficient, *D*, was determined by the pulsed-field gradient spin echo (PFGSE) method [37]. The pulse sequence was used with 16 scans, *τ* of 7.5 ms, and a recycle delay of 6 s. Typical gradient parameters were *Δ* of 7.5 ms, *δ* of 0.5 ms, time between the 90° pulse to the first gradient pulse of 1 ms, and *G* of 1.6 T/m. A double distilled water sample was used for calibration. *D* values of water were taken from [38]. Each reported value is the average of a minimum of ten measurements.

### High-field (HF) ^13^C-NMR relaxometry

Measurements were performed on a BRUKER AVANCE III operating at 150 MHz for ^13^C. Prior to measurement, samples were heated for minimum 10 min and added to a 5-mm NMR tube. For lock signal, a closed 1-mm capillary tube, filled with D_2_O, was added to the sample. The non-spinning samples were allowed to equilibrate inside the instrument for 15 min after reaching the set temperature. Before each measurement, shimming was optimized using automated and manual procedures. The chemical shifts, in parts per millions (ppm), were obtained without reference signal. The spectra were obtained using eight scans and recycle delay of 120 s.

The longitudinal relaxation times, *T*_1_, were measured using the INVREC method [39]. The calculations of *T*_1_ were carried out with the subroutine included in the TOPSPIN 3.2 software package.

^13^C HF-NMR spin–lattice relaxation of a protonated carbon is overwhelmingly dominated by dipole-dipole interactions with the attached protons [28]. *T*_1_ is therefore related to the number of directly bonded hydrogen, N, and the effective correlation time, *τ*_c_, for the rotational movement of the carbon atoms in the object molecule. Thus, *T*_1_ is approximately given in terms of N and 1/*τ*_c_:

2 $$ {\mathrm{T}}_1=\frac{{\mathsf{r}}_{\mathsf{CH}}^{\mathsf{6}}}{N{\hslash}^2{\gamma}_{\mathrm{C}}^2{\gamma}_{\mathrm{H}}^2}\cdot \frac{1}{\tau_{\mathrm{C}}} $$

where *ħ* is Planck’s constant and *γ*_C_ and *γ*_H_ are the gyromagnetic ratios of ^13^C and ^1^H, respectively. Here, *r*_CH_ is the C–H distance, usually about 0.109 nm, and the reciprocal of the effective correlation time, 1/*τ*_c_, represents the magnitude of the segmental rotation for the carbon atom at a different position.

### X-ray methods

XRD and SAXS techniques were used for measuring the short- and long-range spacing between adjacent molecules, respectively.

XRD data was collected on Panalytical Empyrean Powder Diffractometer equipped with position sensitive (PSD) X’Celerator detector using Cu K_α_ radiation (*λ* = 0.154 nm) and operated at 40 kV and 30 mA. The usual Bragg-Brentano *θ*/2θ geometry was employed. *θ*/2θ scans were run during 15 min in a 2θ range of 2–35° with step equal to ~0.0167°.

SAXS measurements were performed on a SAXSLAB GANESHA 300-XL (Skovlunde, Denmark) instrument. Cu K_α_ (*λ* = 0.154 nm) radiation was generated by Genix 3D Cu-source (operated at 47 mV and 0.55 mA) with integrated Monochromator, three pinholes collimation, and two-dimensional Pilatus 300 K detector. The distance between the sample and detector was 350 mm. *q* range was between 0.0012 and 0.067 nm^−1^.

## Additional files

Additional file 1:
**Combined** ^**1**^ **H LF-NMR** ***T***_**2**_ **distributions of 18:3 at increasing degrees of oxidation.** Measurements were performed at 293 K. Plots are denoted A, B, and C by increasing time of natural oxidation. The relative contribution (RC) of the additional peak was found to increase over time, as marked on each plot, while the ratio between RC_1_ and RC_2_ (RC of *P*_1_ and *P*_2_, respectively) was kept unchanged. Additional file 2:
**Combined** ^**1**^ **H LF-NMR** ***T***_**2**_ **distributions of a rapeseed biodiesel sample and its main FAMEs at 313 K.** Plots are arranged by increasing chain length (bottom to top) and increasing degrees of unsaturation. Peaks are assigned to three regions (a, b, c) according to intrinsic *T*_2_s. FAMEs are referred to by their structures.

## Abbreviations

FA: Fatty acid
FAME: Fatty acid methyl ester
HF-NMR: High-field nuclear magnetic resonance
LF-NMR: Low-field nuclear magnetic resonance
PFGSE: Pulsed-field gradient spin echo
RC: Relative contribution
SAXS: Small angle X-ray scattering
XRD: X-ray diffraction
