# Supplementary figures and images for: Liquid-phase characterization of molecular interactions in polyunsaturated and n-fatty acid methyl esters by 1H low-field nuclear magnetic resonance
Source: Biotechnol Biofuels. 2015 Jul 7;8:96. doi: 10.1186/s13068-015-0280-5 (PMC4498500; doi:10.1186/s13068-015-0280-5)

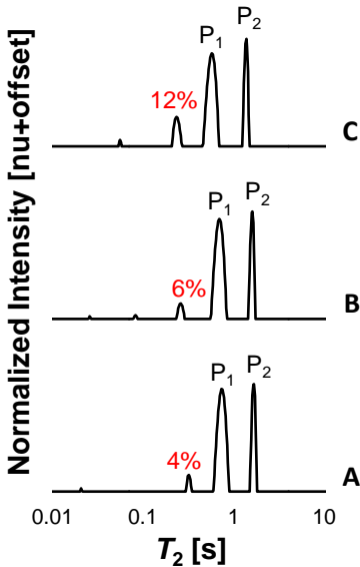

Supplement: Additional file 1: — Combined 1 H LF-NMR T 2 distributions of 18:3 at increasing degrees of oxidation. Measurements were performed at 293 K. Plots are denoted A, B, and C by increasing time of natural oxidation. The relative contribution (RC) of the additional peak was found to increase over time, as marked on each plot, while the ratio between RC1 and RC2 (RC of P 1 and P 2, respectively) was kept unchanged. [file 13068_2015_280_MOESM1_ESM.pdf]

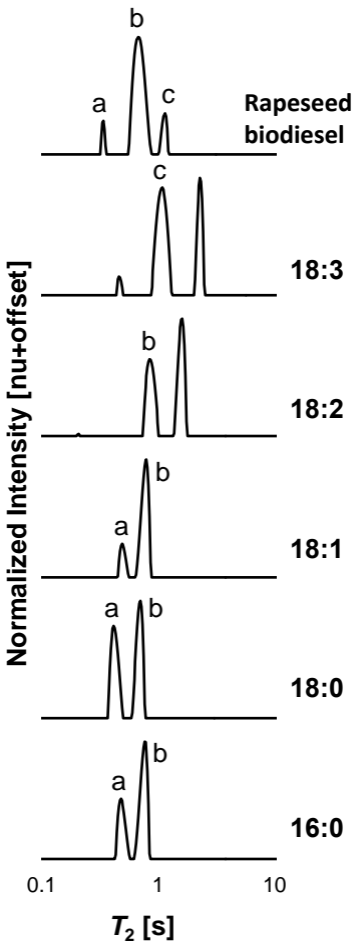

Supplement: Additional file 2: — Combined 1 H LF-NMR T 2 distributions of a rapeseed biodiesel sample and its main FAMEs at 313 K. Plots are arranged by increasing chain length (bottom to top) and increasing degrees of unsaturation. Peaks are assigned to three regions (a, b, c) according to intrinsic T 2s. FAMEs are referred to by their structures. [file 13068_2015_280_MOESM2_ESM.pdf]
